# Synthesis, Characterization, and Antimicrobial Evaluation of Random Poly(ester-Carbonate)s Bearing Pendant Primary Amine in the Main Chain

**DOI:** 10.3390/polym12112640

**Published:** 2020-11-10

**Authors:** Peng Dong, Jing Feng, Sujuan Li, Tingli Sun, Qingshan Shi, Xiaobao Xie

**Affiliations:** Guangdong Provincial Key Laboratory of Microbial Culture Collection and Application, State Key Laboratory of Applied Microbiology Southern China, Guangdong Institute of Microbiology, Guangdong Academy of Sciences, Guangzhou 510070, China; dongpeng@gdim.cn (P.D.); fjing@gdim.cn (J.F.); lsj84324@163.com (S.L.); sunnylilisun@163.com (T.S.)

**Keywords:** cyclic carbonate, primary amine, poly(ester-carbonate)s, antimicrobial activity, low hemolysis and cytotoxicity

## Abstract

Starting from primary amine bearing cyclic carbonate *tert*-butyl-(2-oxo-1,3-dioxan-5-yl) carbamate (TBODC) and caprolactone (CL), amphiphilic poly(caprolactone-*ran*-amino trimethyl carbonate)s (P(CL-*ran*-ATC)s) random copolymers with controlled molecular weight and composition were synthesized via ring opening polymerization (ROP) and deprotection, using stannous octoate (Sn(Oct)_2_) as catalyst and benzyl alcohol (BnOH) as initiator. Therefore, hydrophilic/lipophilic ratio (HLR) of the P(CL-*ran*-ATC)s copolymers can be finely adjusted by the feed ratio of TBODC and CL. The antimicrobial activity against *Staphylococcus aureus* (*S. aureus*) and *Escherichia coli* (*E. coli*) of P(CL-*ran*-ATC)s were proportional to HLR, and P(CL-*ran*-ATC)s presented more vigorous bactericidal activity towards *S. aureus*. The minimum inhibitory concentration (MIC) values of P(CL-*ran*-ATC 50.9%) are 2000 μg mL^−1^ and 3000 μg mL^−1^ for *S. aureus* and *E. coli*. While P(CL-*ran*-ATC 50.9%) exhibited deficient hemolytic activity as 1.41%. In addition, the P(CL-*ran*-ATC)s showed extremely low cytotoxicity towards fibroblast L929 cells.

## 1. Introduction

Antimicrobial peptides (AMPs) are promising antimicrobial agents originating from nature [1,2]. Despite its diverse origins, the basic structure of AMPs is an amphiphilic polypeptide containing cationic amino acid residues [3,4]. Unlike antibiotics that hindered pathogens’ metabolic pathways of [5], AMPs generally kill bacteria by membrane rupture mechanism [6,7]. Hence, it is difficult for the bacteria to develop resistance towards the AMPs. However, toxicity and low yield of the natural AMPs obstruct their application [8]. Additionally, AMPs quickly degraded in physiological environments, resulting in the loss of antibacterial effects [9].

Inspired by the structure of AMPs, amphiphilic polymers bearing cationic segments which are stable to hydrolysis have been widely studied [10,11,12]. The synthetic polymers mimicking AMPs structure showed comparable antimicrobial efficiency towards pathogens. It has been extensively confirmed that the polymers’ hydrophilic/lipophilic ratio (HLR) significantly affected their toxicity and antibacterial ability [13,14,15,16]. Sophisticated control of HLR generally relied on the side chain’s design and the polymer’s main chain. Lipophilic side chain was commonly applied to alter HLR in the AMPs mimicking polymer [3,17]. Many studies have confirmed moderate length of the lipophilic alkyl side chain (5–7 C) was conducive to balance antimicrobial ability and toxicity [18]. Compared to HLR controlled by side chains, HLR controlled by the main chain lacks systematic study [19,20].

The positively charged cations in the AMPs mimics tended to interact with negatively charged bacteria and promoted the lipophilic section of the AMPs mimics penetrating the cell wall causing the death of the bacteria [21]. Amines are widely applied in the AMPs mimics as they present a positive charge under the infection environment [15,22,23]. Compared to quaternary ammonium, which has been generally used in the AMPs mimicking polymers, primary amine showed better antimicrobial property [24,25] while more tendency to cause hemolysis [26].

Introducing biocompatible segments into AMPs mimicking polymers helped relieve toxicity [22,27,28,29,30,31]. Polycaprolactone (PCL) is a biocompatible material [32,33], and its lipophilic character can be used for adjusting HLR of the AMPs mimics [34]. In this research, primary amine bearing cyclic carbonate monomer TBODC was copolymerized with CL via ring opening polymerization (ROP) to get a series of AMP mimicking random copolymers. The protonated P(TBODC-*ran*-ATC)s were obtained via the deprotection process. HLR of P(TBODC-*ran*-ATC)s was adjusted by the initial feed ratio of TBODC to CL. The primary amine carrying precursor polymers and the protonated copolymers were characterized by nuclear magnetic resonance (NMR), gel permeation chromatography (GPC), differential scanning calorimetry (DSC) and thermogravimetric analyses (TGA). Antimicrobial activity of the protonated copolymers was evaluated against Gram-positive *S. aureus* and Gram-negative *E. coli*. Hemolytic activity and cytotoxicity were tested individually. The primary amine functionalized poly(ester-carbonate) with antimicrobial property has various potential applications, including wound dressings and antibiotics carriers.

## 2. Materials and Methods

### 2.1. Materials

Serinol, *di*-tert-butyl dicarbonate (Boc_2_O) were purchased from Meryer Chemical Technology (Shanghai, China). Trifluoroacetic acid (TFA) was purchased from Macklin Biochemical Co. Ltd (Shanghai, China). Ethyl chloroformate (>99.5%) was purchased from Xiya Reagent (Chengdu, China). Stannous octoate (Sn(Oct)_2_, 96%) was obtained from Alfa Aesar (Tianjin, China). Reagents mentioned above were used as received. Caprolactone (ε-CL) was purchased from Aladdin Biochemical Co. Ltd (Shanghai, China). and distilled over CaH_2_ before use. Triethylamine (Et_3_N) was successively treated by *p*-tosyl chloride and CaH_2_ to remove trace amounts of primary amine and H_2_O and stored with 4 Å molecular sieves. Dichloromethane (DCM), tetrahydrofuran (THF), toluene, and benzyl alcohol (BnOH) were dried over CaH_2_ and stored with 4 Å molecular sieves, respectively. All the reagents used in the research were of pure analytical grade.

### 2.2. Measurements

^1^H (400 MHz) and ^13^C NMR (100 MHz) spectra were obtained in deuterated chloroform (CDCl_3_, 99.5 atom % D with 0.03% *v*/*v* TMS), DMSO-*d*_6_ (D, 99.9% with 0.03% *v*/*v* TMS) using a AVANCE III HD 600 spectrometer (Bruker, Berlin, Germany).

Gel permeation chromatography (GPC) measurements were performed on a Waters 1525 binary high-pressure liquid chromatography (HPLC) pump equipped with 3 Ultra Styragel columns and a Waters 2414 refractive index detector (Waters Alliance GPC2000, Waters Corporation, Milford, MA, USA). To characterize the copolymers, THF was used as an eluent at a flow rate of 1 mL/min at 40 °C. The number-averaged molecular weight (*M*_n_) and molar mass distribution (*Ð*) were calculated using Waters GPC Software, and narrowly dispersed polystyrenes (PS) were employed as calibration standards.

The copolymers’ thermal properties were determined on a TA Instruments Q10 Differential Scanning Calorimeter (DSC, TA Instruments, New Castle, DE, USA). The enthalpy (cell constant) and temperature were calibrated by running high-purity indium and gallium standards under conditions identical to those used to measure the samples.

Zeta potential values were measured on Malvern Zetasizer Pro with He-Ne lamp at 633 nm (Malvern Instruments, Malvern, UK).

High-resolution mass spectrometry (HRMS) was determined on Waters G2-XS Qtof (Waters Corporation, Milford, MA, USA).

Thermogravimetric analyses (TGA) were carried out using an STA 449 F3 Jupiter^®^ simultaneous thermogravimetry-differential scanning calorimetry analyzer (NETZSCH-Gerätebau GmbH, Selb/Upper Franconia, Germany).

Organic elemental analyses (OEA) were conducted using a Vario EL cube analyzer (Elementar Analysensysteme GmbH, Frankfurt am Main, Germay).

### 2.3. Experimental Methodology

#### 2.3.1. Synthesis of Cyclic Carbonates Bearing Potential Primary Amino Groups

TBODC was synthesized according to the previous report with some modifications [35]. Firstly, serinol (10.0 g, 11 mmol) was dissolved in 150 mL anhydrous ethanol, and Boc_2_O (30.3 mL, 13 mmol) was added dropwise. The mixture was stirred overnight at room temperature. Then ethanol was removed by rotary evaporation, and the residual was crystallized by a mixture of ethyl acetate/petroleum ether (*v*/*v* = 1/1). The white crystal was filtered to obtain intermediate *tert*-butyl (1,3-dihydroxypropan-2-yl)carbamate (Boc-serinol, 17.63 g). Yield: 83.5%. mp 90.8–92.7 °C. ^1^H NMR (400 MHz DMSO-*d*_6_, δ): 6.32 (s, 1H, -N*H*COO-), 4.53–4.45 (*t*, 2H, -CH_2_O*H*), 3.43–3.33 (dm, 5H, -C*H*_2_CH- and -CH_2_C*H*-), 1.37 (s, 9H, -C*H*_3_).

Boc-serinol (15 g, 78 mmol) and ethyl chloroformate (14.9 mL, 156 mmol) were completely dissolved in 200 mL anhydrous THF. The mixture was cooled to 0 °C. Then, Et_3_N (27.1 mL, 195 mmol) was added dropwise, and white precipitation formed. The reaction was to lift to room temperature for another hour after the complete addition of Et_3_N. After filtration of the white precipitate, the filtrate was condensed by rotary evaporation, and then petroleum ether was added to obtain TBODC as white flakes (TBODC, 6.94 g). Yield: 58.5%. mp. 139.0–140.8 °C. HRMS: *m/z* calcd for C_9_H_16_NO_5_ 208.1028, found 208.1019. ^1^H NMR (400 MHz, DMSO-*d*_6_, δ): 7.60 (s, 1H, -CON*H*-), 4.52 (dd, *J* = 3.5, 7.0 Hz, 2H, -OC*H*_2_-), 4.24 (dd, *J* = 3.5, 7.0 Hz, 2H, -OC*H*_2_-), 3.92 (s, 1H, -CH_2_C*H*-), 1.40 (s, 9H, -C*H*_3_). ^13^C NMR (100 MHz, DMSO-*d*_6_, δ): 155.38, 153.58, 78.55, 66.95, 41.80, 28.11. Anal. Calcd for C_9_H_16_NO_5_: C, 49.53; H, 7.39; N, 6.42; O, 36.66. Found: C, 49.27; H, 7.52; N, 6.63; O, 36.58.

#### 2.3.2. Typical Synthesis of Random Copolymer Poly(ε-caprolactone-ran-tert-butyl (2-oxo-1,3-dioxan-5-yl)carbamate) (P(CL-ran-TBODC))

Synthesis of P(CL-*ran*-TBODC) was conducted with the standard Schlenk technique under an atmosphere of nitrogen unless stated otherwise. For instance, 0.2 g TBODC (0.92 mmol), 920 μL CL (8.28 mmol), 9.6 μL BnOH (0.092 mmol), 14.9 μL SnOct_2_ (0.046 mmol) were added into a Schlenk flask. Then, 5 mL dry toluene was added into the flask, and the freezing–thawing cycle was conducted three times to remove oxygen and moisture. After the flask was charged with N_2_, polymerization was conducted at 100 °C. After polymerization for 24 h, ^1^H NMR monitored that conversion of both TBODC and CL exceeded 90%. Then the polymerization was stopped by cooling to room temperature, and the crude product was immediately precipitated by cold ethanol. Then, the crude polymer was dissolved and precipitated twice by THF and petroleum ether. The polymer was collected by filtration and dried in a vacuum oven for two days until constant weight. The polymer yield was over 90%.

#### 2.3.3. Preparation of Poly(ester-carbonate)s Bearing Primary Amine

Deprotection of P(CL-*ran*-TBODC) was conducted as follows: 0.5 g P(CL-*ran*-TBODC) was completely dissolved in DCM. Then trifluoroacetic acid (TFA) was added dropwise at 0 °C. The deprotection was conducted for one hour to remove Boc groups. Then, deprotected polymer P(caprolactone-*ran*-amino trimethyl carbonate)s (P(CL-*ran*-ATC)s) were precipitated by diethyl ether. The product was dissolved-precipitated twice and dried in a vacuum oven till constant weight.

#### 2.3.4. Antimicrobial Activity

Bacterial growth inhibition was evaluated by minimum inhibitory concentration (MIC) assay via the broth microdilution method. The MIC values of P(CL-*ran*-ATC)s were determined against *S. aureus* (ATCC No. 6538, Gram-positive) and *E. coli* (ATCC No. 8739, Gram-negative). Bacterial colonies were inoculated in Luria broth (LB) and left to grow overnight at 37 °C until the exponential phase while being shaken continuously. The overnight cultures of bacteria were adjusted to an optical density of 0.1 at 600 nm (OD600), equal to 10^8^ CFU/mL. After washing in phosphate-buffered saline (PBS; NaH_2_PO_4_, K_2_HPO_4_, NaCl), the bacteria were diluted by Mueller–Hinton broth (MHB) to obtain a stock solution of 10^6^ CFU/mL. The P(CL-*ran*-ATC)s were then diluted using the PBS buffer solution to bring the copolymers to the desired concentration range. After that, 40 μL of bacterial stock solution was added to each dilution and incubated at 37 °C for 24 h. The OD600 was then measured using a 96 well microplate reader. The minimum inhibitory concentrations (MIC) were later reported as the concentration at which no bacterial growth was observed.

#### 2.3.5. Hemolysis

Hemolysis caused by P(CL-*ran*-ATC)s was determined by hemoglobin release assay using the same P(CL-*ran*-ATC)s stock solutions used in the MIC test. Then, 1 mL of 4% (*v*/*v*) rabbit red blood cells (RBCs) was centrifuged at 1000 rpm for 5 min and after that washed with PBS solution of pH 7.4. The supernatants were then removed by pipetting. The RBCs were further washed with PBS twice. The RBCs were then diluted using PBS to provide 1% (*v*/*v*) RBCs assay stock. In a 96-well microplate, 90 μL of 1% (*v*/*v*) RBCs assay stock was mixed with 10 μL of the copolymer dilution. PBS with 5% (*v*/*v*) of DMSO was used as a negative control, while PBS with 0.1% (*v*/*v*) of Triton X-100 was used as a positive control. The microplate was sealed using a parafilm and incubated in an orbital shaker at a temperature of 37 °C at 180 rpm for 60 min. The microplate was then centrifuged at 1000 rpm for 10 min. In another microplate, 10 μL of supernatant was diluted in 90 μL of PBS. The absorbance at 415 nm was recorded using a microplate reader. Hemolysis fraction was then calculated using Equation (1).

(1) $$H=\frac{OD_{415s}-OD_{415n}}{OD_{415p}-OD_{415n}}$$

where *OD*_415*s*_ is the absorbance of the test well, *OD*_415*n*_ is the absorbance of negative control well, and *OD*_415*p*_ is the absorbance of positive control well.

The assay was performed in triplicates, with three replicates for each independent experiment.

#### 2.3.6. Cyto-Compatibility

The cytotoxicity was checked on the first, second, and fourth days of culture using a cell counting kit-8 (CCK-8) assay. Fibroblast L929 suspension of 100 μL was seeded at a density of 1 × 10^3^ cells/well and was pre-incubated at 37 °C for 4 h in a 5% CO2 incubator. The P(CL-*ran*-ATC)s copolymers were sterilized through filtration and diluted by PBS. Then, 10 μL of each diluted P(CL-*ran*-ATC)s copolymer was transferred to the cell seeding 96-well plate. The culture medium was changed every other day. Then, 10 μL of a CCK-8 solution was added to each well and incubated for 2 h according to the manufacturer’s instructions. After 2 h of incubation, the absorbance was measured at a wavelength of 450 nm using a microplate reader (Multiskan GO, Thermo Scientific, Waltham, MA, USA). The cell viability was calculated using Equation (2):

(2) $$\mathrm{The}\;\mathrm{cell}\;\mathrm{viability}=\frac{A_{s}-A_{b}}{A_{c}-A_{b}}\times 100\%$$

where *A_s_* is the absorbance of the test well, *A_c_* is the absorbance of negative control well, and *A_b_* is the absorbance of blank control well.

The assay was performed in triplicates, with three replicates for each independent experiment.

## 3. Results and Discussion

### 3.1. Synthesis of Cyclic Carbonate TBODC

**Scheme 1:**
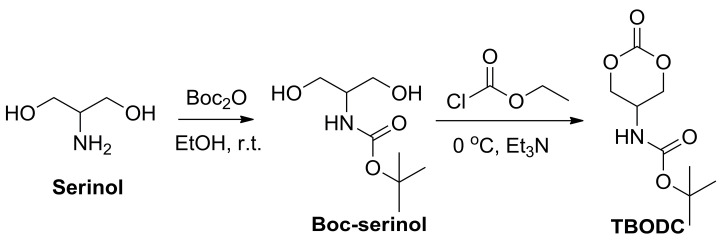
Synthesis of Boc-serinol and carbonate *tert*-butyl-(2-oxo-1,3-dioxan-5-yl) carbamate (TBODC).

TBODC, a cyclic carbonate monomer bearing potential primary amine groups, was synthesized by a two-step reaction starting from serinol (Scheme 1). Primary amine in serinol was protected by di-*tert*-butyl dicarbonate with high yield. The protection prevented interference with the ring closure reaction. The Boc-serinol intermediate was cyclocarbonated by ethyl chloroformate in the presence of triethylamine at 0 °C in THF. Finally, the cyclic carbonate TBODC was obtained with an overall yield of 50%. The structures of TBODC were characterized by ^1^H NMR and ^13^C NMR (Figures S1 and S2). Both appearance of the Boc signal and the signal shift of methylene indicated a successful synthesis of TBODC. In addition, the HRMS spectra and elemental analysis results confirmed the successful preparation of TBODC monomer.

### 3.2. Random Copolymerization of CL and TBODC

**Scheme 2:**
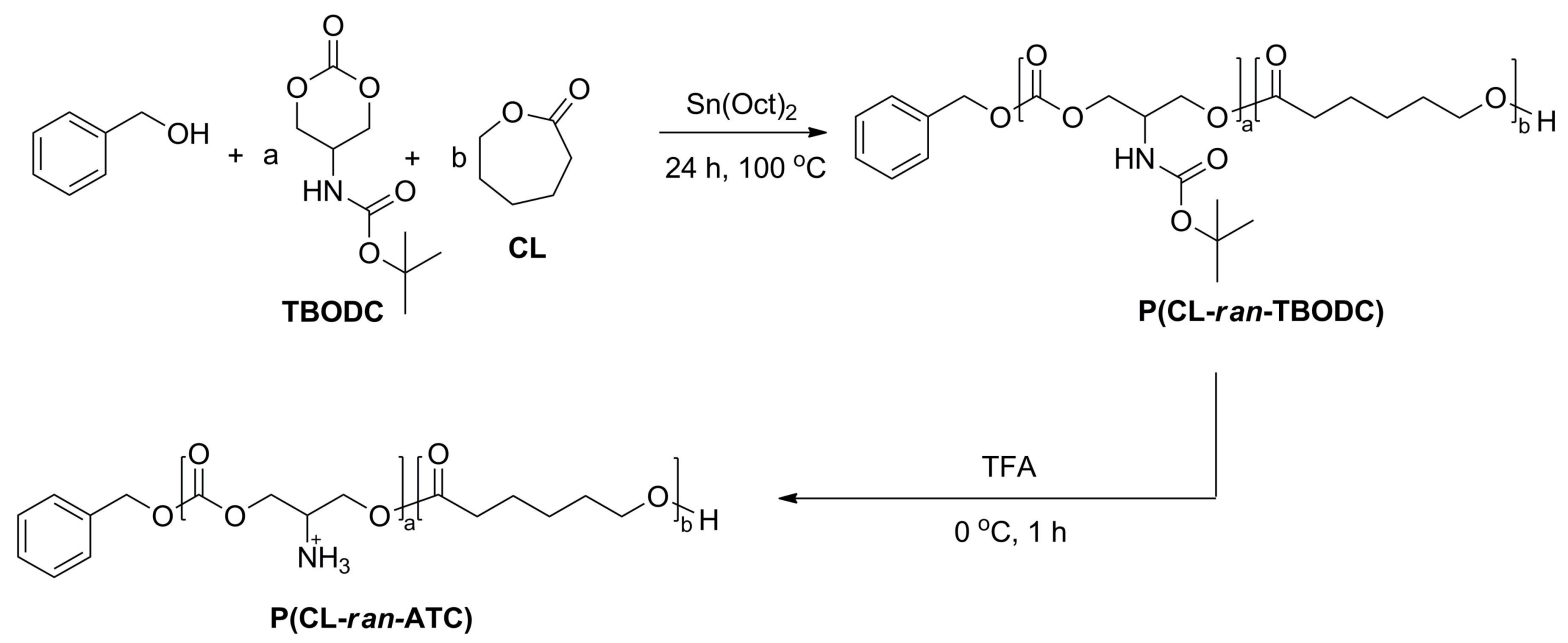
Synthesis of random copolymer P(CL-*ran*-TBODC) and deprotection.

The semi-crystalline structure decreased the solubility of PCL [36,37,38]. Here, random copolymerization of TBODC and ε-CL was conducted to improve the copolymer’s solubility by disrupting the semi-crystalline region. The copolymerization was done in toluene using benzyl alcohol as initiator and SnOct_2_ as catalyst (Scheme 2).

**Figure 1:**
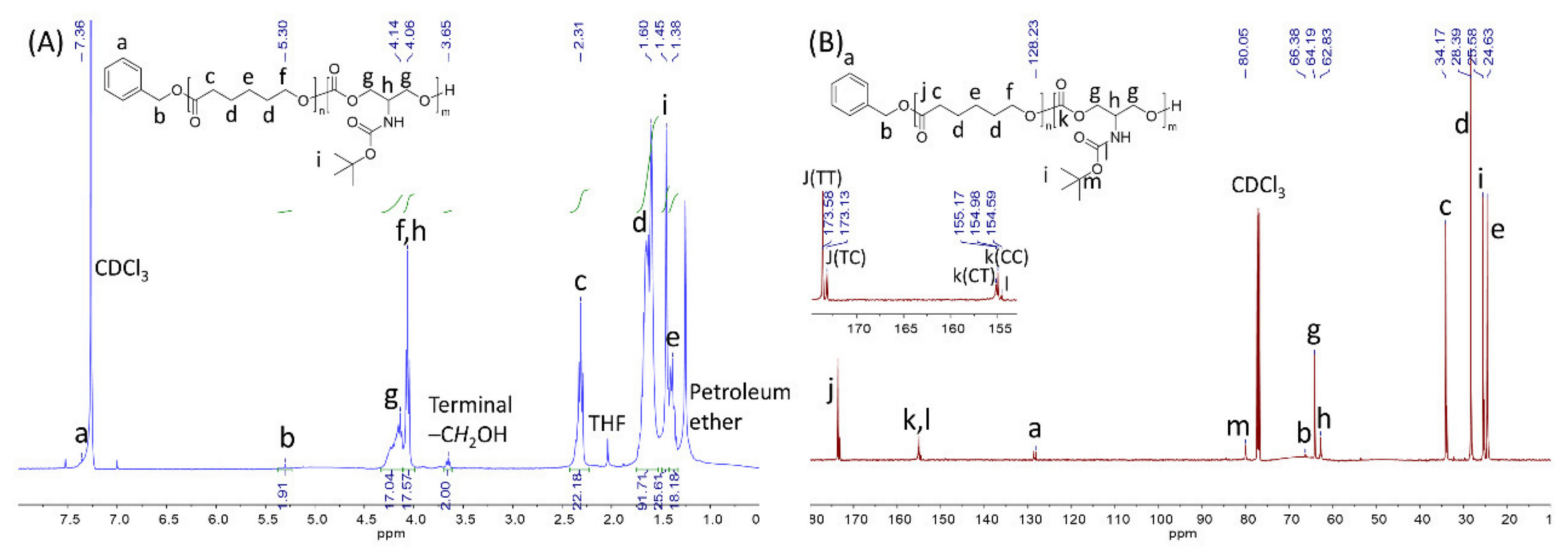
(**A**) ^1^H NMR spectrum of P(CL-*ran*-TBODC 22.8%) (400 MHz, CDCl_3_, 25 °C); (**B**) ^13^C NMR spectrum of P(CL-*ran*-TBODC 22.8%). Signals labeled TT, TC, CT, CC correspond to TBODC-TBODC, TBODC-CL, CL-TBODC, and CL-CL dyads (100 MHz, CDCl_3_, 25 °C).

**Figure 2:**
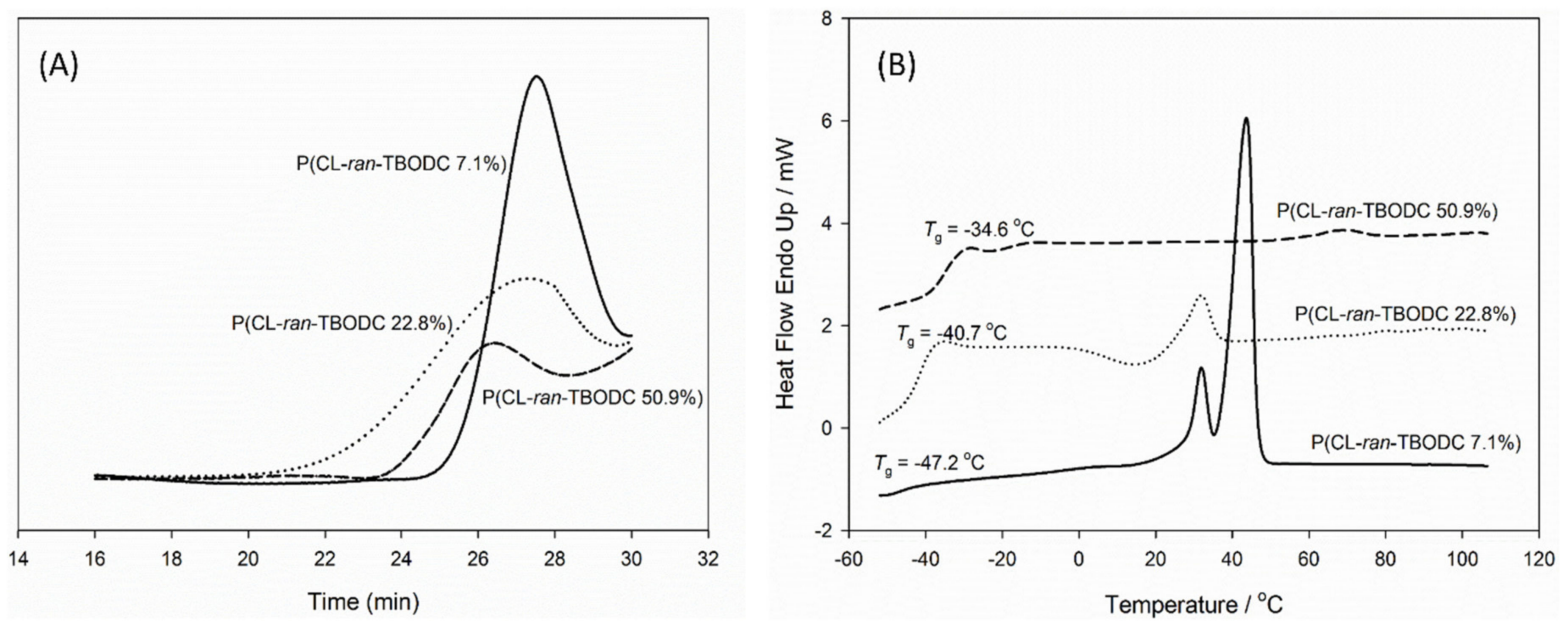
(**A**) Gel permeation chromatography (GPC) curves of P(CL-*ran*-TBODC), (**B**) second heating scan of DSC traces of P(CL-*ran*-TBODC).

The comonomer to initiator ratio was set at 100/1, and TBODC in monomer feed varied from 10%, 25%, to 50% (Table 1, entries 1–3). After 24 h, the polymerizations were stopped, and the conversion of both TBODC and ε-CL exceeded 90% (Figure S3). The initiator’s methylene hydrogen signal was selected to calculate the number average molecular weight (*M*_n_) of the copolymers. P(CL-*ran*-TBODC X%) was short for the copolymers of TBODC and ε-CL, where X% stood for the percentage of TBODC segment in the copolymer. As shown in Table 1, TBODC ratio in the copolymers almost coincided with the monomer feed ratio, which suggested fine control of SnOct_2_ over the copolymerization. In addition, the protection of primary amine omitted possible interference on the ROP reaction. A typical ^1^H NMR spectrum of P(CL-*ran*-TBODC 22.8%) (Table 1, entry 2) is shown in Figure 1A. Signal of -OC*H*_2_- shifted from 4.55 ppm to 4.16 ppm indicated ring opening of TBODC. Furthermore, the presence of signals corresponding to PCL suggested that successful copolymerization of TBODC and CL. ^13^C NMR spectra also confirmed the synthesis of P(CL-*ran*-TBODC). As displayed in Figure 1B, peaks corresponding to P(CL-*ran*-TBODC 22.8%) were assigned. Signals of the carbonyl carbon of ester (j, 178.50–175.50 ppm) and carbonate (k, 138.30–136.40 ppm) split into two peaks as shown in the insert figure in Figure 1B. The signals were labeled as TT, TC, CT, and CC corresponding to TBODC-TBODC, TBODC-CL, CL-TBODC, and CL-CL dyads. The appearance of four dyads confirmed the preparation of random copolymers [39]. GPC curves indicated successful random copolymerizations were accomplished as uni-peaks showed up (Figure 2A). Meanwhile, low molar mass dispersities (*Đ* = 1.26 to 1.58) of the copolymer also revealed fine control of the copolymerization. DSC result confirmed that including TBODC segments can alter crystallinity of the copolymer. As shown in Figure 2B, the melting peak disappeared with an increase of TBODC ratio in the copolymer. In addition, the copolymer’s glass transition temperature (*T*_g_) increased with a rise in TBODC ratio in the copolymer. It could ascribe to the bulky Boc groups hindered the chain segment’s movement, which caused a higher *T*_g_.

### 3.3. Deprotection of P(CL-ran-TBODC)

**Figure 3:**
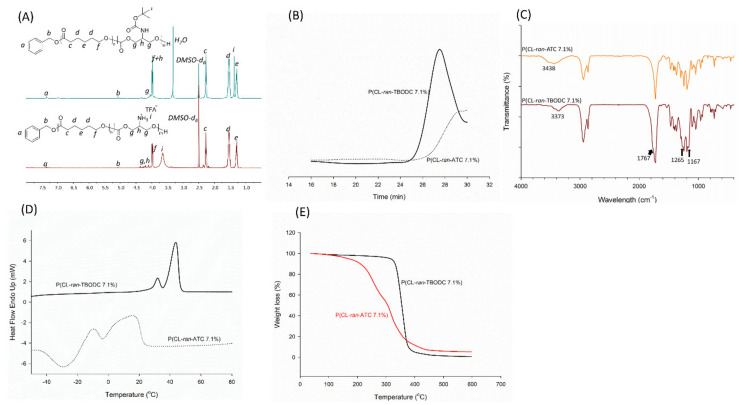
(**A**) ^1^H NMR spectra of P(CL-*ran*-ATC 7.1%) and P(CL-*ran*-TBODC 7.1%) (DMSO-*d*_6_, 400 MHz); (**B**) GPC curves of P(CL-*ran*-ATC 7.1%) and P(CL-*ran*-TBODC 7.1%); (**C**) FT-IR spectra of P(CL-*ran*-ATC 7.1%) and P(CL-*ran*-TBODC 7.1%); (**D**) DSC traces of P(CL-*ran*-ATC 7.1%) and P(CL-*ran*-TBODC 7.1%); (**E**) TGA traces of P(CL-*ran*-ATC 7.1%) and P(CL-*ran*-TBODC 7.1%).

Boc groups were removed by TFA to endow hydrophilicity and positive charge to the copolymers. ^1^H NMR was used to carefully monitor the deprotection progress to prevent excessive degradation of the copolymer. It was confirmed deprotection for 1 h was enough to remove Boc (Figure S4). The deprotected copolymer was obtained by adding diethyl ether, and the signal of Boc groups in carbonate segments almost disappeared. FT-IR results also confirmed the total removal of the Boc groups (Figure 3C). Peaks corresponding to Boc groups (1767 cm^−1^, 1265 cm^−1^, 1167 cm^−1^) disappeared after deprotection. The peak of primary amine showed up at 3438 cm^−1^. The deprotected copolymers were named after P(CL-*ran*-ATC x%), where ATC stood for carbonate segments after deprotection and x meant for carbonate ratio in the deprotected copolymers. Meanwhile, x was also exhibited as HLR in this study. Take deprotection of P(CL-*ran*-TBODC 7.1%) as an example, the methyl signal at 1.37 ppm disappeared and the signal at 3.65 ppm corresponding to freed amine shown up (Figure 3A). The NMR signal change indicated successful deprotection of Boc groups. Both NMR integration and uni-peak of GPC trace (Figure 3B) suggested no severe degradation occurred during deprotection. Compared to its precursor polymer P(CL-*ran*-TBODC 7.1%), an exothermic peak showed up at −29.01 °C in the second heating round of P(CL-*ran*-ATC 7.1%) (Figure 3C). As the bulky Boc groups were removed, the deprotected copolymer’s main chain was susceptible to undergoing a cold crystallization process. Hence, the melting point of P(CL-*ran*-ATC 7.1%) also decreased. TGA analysis confirmed that P(CL-*ran*-ATC 7.1%) tended to undergo pyrolytic degradation compared to its precursor P(CL-*ran*-TBODC 7.1%) (Figure 3D). It was due to the bare primary amine was not stable and can cause degradation of the copolymer.

With the increase of carbonate content in the copolymer, P(CL-*ran*-ATC)’s solubility in DI water increased after the deprotection process. Notably, P(CL-*ran*-ATC 50.9%) formed a homogeneous aqueous solution. P(CL-*ran*-ATC 50.9%) solution showed 38.3 ± 1.2 mV Zeta potential, while Zeta potential P(CL-*ran*-ATC 22.8%) was 25.8 ± 1.8 mV. Zeta potential determination of P(CL-*ran*-ATC 7.1%) was failed as poor solubility of P(CL-*ran*-ATC 7.1%) in DI water. Surprisingly, PATC showed a Zeta potential of 7.7 ± 0.7 mV, which was lower than P(CL-*ran*-ATC 50.9%) and P(CL-*ran*-ATC 22.8%). Different main chain structure of P(CL-*ran*-ATC) and homo-PATC might be responsible for the Zeta potential determination.

The deprotected primary amine in P(CL-*ran*-ATC)s tended to form -NH_3_^+^ammonium ions under physiological condition, which is responsible for amphiphilicity and positive Zeta potential of P(CL-*ran*-ATC)s. Principally, P(CL-*ran*-ATC)s were structural analogs of antimicrobial peptides (AMPs). In the following study, the relationship between the structure of P(CL-*ran*-ATC)s and their antimicrobial property was studied.

### 3.4. Antimicrobial Property, Hemolytic Activity, and Cytotoxicity

The antimicrobial property of P(CL-*ran*-ATC)s was studied by minimum inhibitory concentrations (MIC) test via the microdilution method. *S. aureus* (ATCC No. 6538) and *E. coli* (ATCC No. 8739) were applied as model Gram-positive and Gram-negative bacteria. P(CL-*ran*-ATC)s was dissolved into 5000 μg mL^−1^, and the concentration subsequently diluted until 1 μg mL^−1^. As demonstrated in Table 2, MICs of P(CL-*ran*-ATC)s generally decreased with the increase of HLR of the copolymers. The results showed that the increase in the content of hydrophilic carbonate segments in the random copolymer P(CL-*ran*-ATC)s is conducive to enhancing the antimicrobial property. P(CL-*ran*-ATC 7.1%) showed no antibacterial effect even at 5000 μg mL^−1^. It should ascribe to the low solubility of P(CL-*ran*-ATC 7.1%).

**Figure 4:**
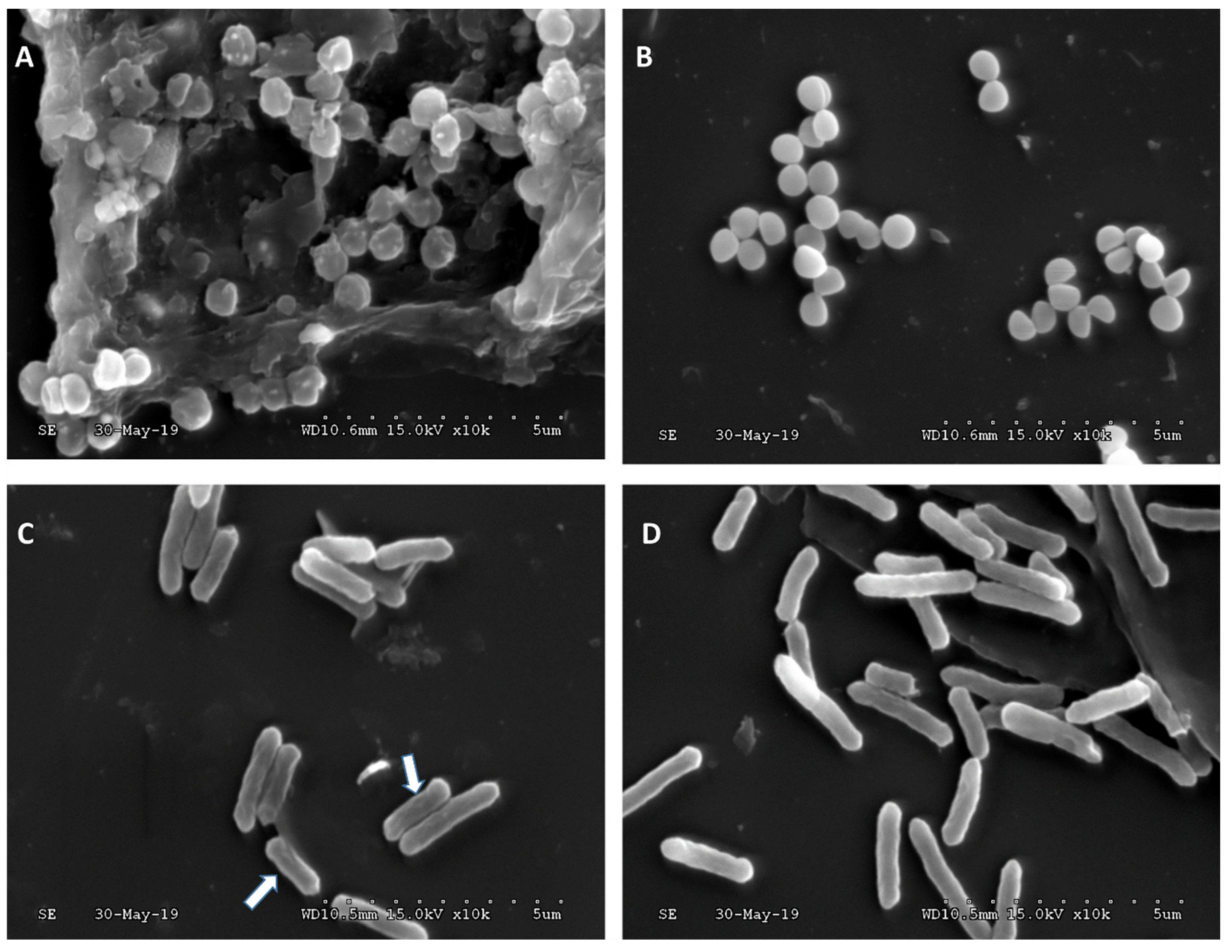
SEM images of *S. aureus* incubated (**A**) in the presence of P(CL-*ran*-ATC 50.9%) (3000 μg mL^−1^), (**B**) in the absence of polymer; *E. coli* incubated (**C**) in the presence of P(CL-ran-ATC 50.9%) (3000 μg mL^−1^), (**D**) in the absence of polymer. The scale bar was 5 μm.

As confirmed by MIC, the P(CL-*ran*-ATC) copolymers showed a relatively more substantial antimicrobial effect towards Gram-positive *S. aureus*. The results are consistent with previously reported that amino-bearing polyesters have a better bactericidal effect on Gram-positive bacteria [30,31]. SEM images also confirmed the results of MIC (Figure 4). *S. aureus* significantly shrank after treatment with P(CL-*ran*-ATC 50.9%)s and rupture can be observed. *E. coli* showed a similar trend, but slight shrinkage was observed (arrow in Figure 4C). As confirmed by SEM, the morphological change of bacterial membrane accorded with the membrane rupture mechanism after treatment with AMPs.

Surprisingly, homopolymer PATC presented a weaker antibacterial effect than P(CL-*ran*-ATC 22.8%) and P(CL-*ran*-ATC 50.9%). It reminded the lipophilic segment is necessary for promoting antimicrobial effect for poly(ester-carbonate)s. In addition, MICs of P(CL-*ran*-ATC)s were high when they were compared with other antimicrobial poly(ester-carbonate)s [40,41]. Cationic centers distributed on the main chain and the random structure of P(CL-*ran*-ATC)s may hinder interaction between the copolymer and the bacteria, which led to a high MIC value of P(CL-*ran*-ATC)s.

Previous literature reported that polymers containing primary amine groups are prone to hemolysis [27]. Here, rabbit red blood cells (rRBCs) were used to evaluate the hemolytic activity of P(CL-*ran*-ATC)s (Table 2). The rRBCs remained a complete structure even when the concentration of P(CL-*ran*-ATC)s reached 4000 μg mL^−1^. The hemolysis rate was lower than 2.5% during the whole test. Moreover, hemolysis slightly increased with a decrease of HLR of P(CL-*ran*-ATC)s. The results indicated that the increase of hydrophobic PCL segments caused a decrease of HLR and slightly promoted hemolysis.

**Figure 5:**
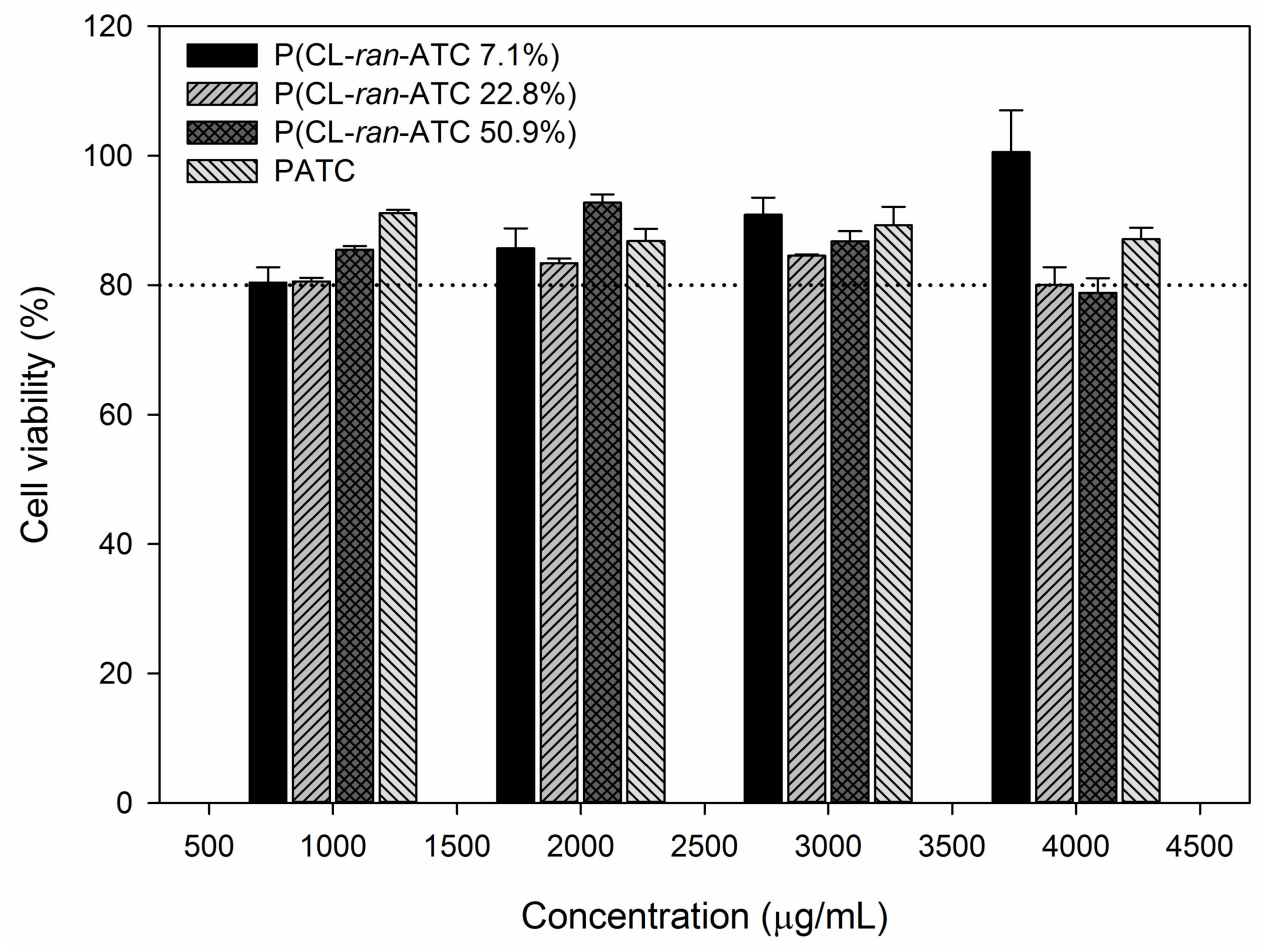
Cytotoxicity of P(CL-*ran*-ATC)s against fibroblast L929 cells.

To further characterize the cytotoxicity of P(CL-*ran*-ATC)s, fibroblast L929 was co-cultivated with P(CL-*ran*-ATC)s. After culture for three days, Cell Counting Kit-8 (CCK-8) was used. As shown in Figure 5, cells proliferated well in all groups, indicating that the cytotoxicity of P(CL-*ran*-ATC)s can be ignored.

## 4. Conclusions

In conclusion, primary bearing cyclic carbonate TBODC was copolymerized with CL to form random copolymers via ring opening polymerization. The random copolymers turned amphiphilic after the removal of Boc protecting groups. Hydrophilic/lipophilic ratio (HLR) of the protonated poly(ester-carbonate)s can be adjusted by the feed ratio of TBODC and CL. P(CL-*ran*-ATC 50.9%) exhibited higher antimicrobial activity than P(CL-*ran*-ATC 7.1%) and P(CL-*ran*-ATC 22.8%) due to higher HLR. All the P(CL-*ran*-ATC)s exhibited low hemolytic activity, and the hemolysis rate slightly increased with a decrease of HLR. P(CL-*ran*-ATC)s were proved to be free of cytotoxicity. Further study on block copolymer of TBODC and CL is under investigation. We anticipate that by altering polymer’s structure, the antibacterial ability of P(CL-*co*-ATC)s can be improved.

## Figures and Tables

**Table 1:** Ring opening polymerization of TBODC and ε- caprolactone (CL) catalyzed by SnOct_2_^a^.

Entry	*f*_TBODC/ε-CL_^b^	^1^H NMR		GPC ^e^		DSC	
		*F* ^c^	*M*_n_/kDa ^d^	*M*_n_/kDa	*Đ*	*T*_g_/°C	*T*_m_/°C
1	10/90	7.1	8.3	8.7	1.26	−47.15	49.54
2	25/75	22.8	10.7	14.9	1.58	−40.73	36.53
3	50/50	50.9	3.9	17.7	1.30	−34.59	N/A
4	100/0	N/A	4.8	3.3	1.10	48.45	N/A
5	0/100	N/A	12.2	8.3	1.40	/^f^	63.50

^a^ Polymerization conditions: [comonomer]/[BnOH]/[SnOct_2_] = 100/1/0.5, 100 °C, 24 h, 1 M in toluene; ^b^ feed ratio of TBODC to ε-CL in comonomer; ^c^ TBODC segment ratio in copolymers which is determined by ^1^H NMR via 0.5 × *I*_4.14ppm_/(0.5 × *I*_4.14ppm_ + 0.5 × *I*_2.31ppm_); ^d^ molecular weight calculated by ^1^H NMR via 0.5 × *I*_4.14 ppm_ × 217.22 + 0.5 × *I*_2.31 ppm_ × 114.14 + 107.41; ^e^ THF used as elution; ^f^ *T*_g_ of PCL was not detected due to cooling depth limitation.

**Table 2:** Antimicrobial (MIC), hemolytic activities (HC_4000_), and Zeta potential of the polycationic copolymers.

Polymer	HLR ^a^	MIC (μg mL^−1^)		Hemolytic Activity (H_4000_) ^b^	Zeta Potential (mV)
		*S. aureus* (Gram +)	*E. coli* (Gram −)		
P(CL-*ran*-ATC 7.1%)	7.1	NA ^c^	NA ^c^	2.16%	/^d^
P(CL-*ran*-ATC 22.8%)	22.8	3500	4000	1.53%	25.8 ± 1.8
P(CL-*ran*-ATC 50.9%)	50.9	2000	3000	1.41%	38.3 ± 1.2
PATC	/	4000	NA ^c^	0.74%	7.7 ± 0.7

^a^ Hydrophilic/lipophilic ratio (HLR) is protonated carbonate segment ratio in P(CL-*ran*-ATC). ^b^ H_4000_ is the hemolysis percentage at a concentration of 4000 μg mL^−1^. ^c^ NA indicates that no antibacterial effect was observed even at 5000 μg mL^−1^. ^d^ Undetectable.

## Supplementary Materials

The following are available online at https://www.mdpi.com/2073-4360/12/11/2640/s1, Figure S1: ^1^H NMR spectra of serinol (a), Boc-serinol (b) and TBODC(c)(DMSO-*d*_6_, 400 MHz); Figure S2. ^13^C NMR spectrum of TBODC (DMSO-*d*_6_, 100 MHz); Figure S3. Investigation of copolymerization time of TBODC and CL; Figure S4. ^1^H NMR spectra of P(CL-*ran*-TBODC 50.9%) after deprotection for certain time.

## References

[1] Rathinakumar R., Walkenhorst W.F., Wimley W.C. (2009). Broad-spectrum antimicrobial peptides by rational combinatorial design and high-throughput screening: The importance of interfacial activity. J. Am. Chem. Soc..

[2] Barman S., Konai M.M., Samaddar S., Haldar J. (2019). Amino Acid Conjugated Polymers: Antibacterial Agents Effective against Drug-Resistant Acinetobacter baumannii with No Detectable Resistance. ACS Appl. Mater. Interfaces.

[3] Yang Y., Cai Z., Huang Z., Tang X., Zhang X. (2018). Antimicrobial cationic polymers: From structural design to functional control. Polym. J..

[4] Carmona-Ribeiro A.M., de Melo Carrasco L.D. (2013). Cationic antimicrobial polymers and their assemblies. Int. J. Mol. Sci..

[5] Gottlieb D., Shaw P.D. (1967). Antibiotics Volume I Mechanism of Action.

[6] Epand R.F., Mowery B.P., Lee S.E., Stahl S.S., Lehrer R.I., Gellman S.H., Epand R.M. (2008). Dual Mechanism of Bacterial Lethality for a Cationic Sequence-Random Copolymer that Mimics Host-Defense Antimicrobial Peptides. J. Mol. Biol..

[7] Yeaman M.R., Yount N.Y. (2003). Mechanisms of Antimicrobial Peptide Action and Resistance. Pharmacol. Rev..

[8] Ng V.W.L., Ke X., Lee A.L.Z., Hedrick J.L., Yang Y.Y. (2013). Synergistic co-delivery of membrane-disrupting polymers with commercial antibiotics against highly opportunistic bacteria. Adv. Mater..

[9] Ageitos J.M., Sánchez-pérez A., Calo-mata P., Villa T.G. (2017). Antimicrobial peptides (AMPs): Ancient compounds that represent novel weapons in the fight against bacteria. Biochem. Pharmacol..

[10] Muñoz-Bonilla A., Fernández-García M. (2015). The roadmap of antimicrobial polymeric materials in macromolecular nanotechnology. Eur. Polym. J..

[11] Lee A.L.Z., Ng V.W.L., Wang W., Hedrick J.L., Yang Y.Y. (2013). Block copolymer mixtures as antimicrobial hydrogels for biofilm eradication. Biomaterials.

[12] Kuroki A., Sangwan P., Qu Y., Peltier R., Sanchez-Cano C., Moat J., Dowson C.G., Williams E.G.L., Locock K.E.S., Hartlieb M. (2017). Sequence Control as a Powerful Tool for Improving the Selectivity of Antimicrobial Polymers. ACS Appl. Mater. Interfaces.

[13] Pinazo A., Petrizelli V., Bustelo M., Pons R., Vinardell M.P., Mitjans M., Manresa A., Perez L. (2016). New cationic vesicles prepared with double chain surfactants from arginine: Role of the hydrophobic group on the antimicrobial activity and cytotoxicity. Colloids Surfaces B Biointerfaces.

[14] Zhao R., Wang H., Ji T., Anderson G., Nie G., Zhao Y. (2015). Biodegradable cationic epsilon-poly-L-lysine-conjugated polymeric nanoparticles as a new effective antibacterial agent. Sci. Bull..

[15] Xu Q., Yang C., Hedrick J.L., Yang Y.Y. (2016). Antimicrobial silica particles synthesized via ring-opening grafting of cationic amphiphilic cyclic carbonates: Effects of hydrophobicity and structure. Polym. Chem..

[16] Yuin Z., Cheng J., Huang Y., Xu K., Ji Z., Fan W. (2014). Biomaterials Effect of stereochemistry, chain length and sequence pattern on antimicrobial properties of short synthetic β -sheet forming peptide amphiphiles. Biomaterials.

[17] Punia A., He E., Lee K., Banerjee P., Yang N.L. (2014). Cationic amphiphilic non-hemolytic polyacrylates with superior antibacterial activity. Chem. Commun..

[18] Engler A.C., Tan J.P.K., Ong Z.Y., Coady D.J., Ng V.W.L., Yang Y.Y., Hedrick J.L. (2013). Antimicrobial polycarbonates: Investigating the impact of balancing charge and hydrophobicity using a same-centered polymer approach. Biomacromolecules.

[19] Cuthbert T.J., Hisey B., Harrison T.D., Trant J.F., Gillies E.R., Ragogna P.J. (2018). Surprising Antibacterial Activity and Selectivity of Hydrophilic Polyphosphoniums Featuring Sugar and Hydroxy Substituents. Angew. Chem. Int. Ed..

[20] Palermo E.F., Lienkamp K., Gillies E.R., Ragogna P.J. (2019). Antibacterial Activity of Polymers: Discussions on the Nature of Amphiphilic Balance. Angew. Chem. Int. Ed..

[21] Siedenbiedel F., Tiller J.C. (2012). Antimicrobial polymers in solution and on surfaces: Overview and functional principles. Polymers.

[22] Pascual A., Tan J.P.K., Yuen A., Chan J.M.W., Coady D.J., Mecerreyes D., Hedrick J.L., Yang Y.Y., Sardon H. (2015). Broad-Spectrum Antimicrobial Polycarbonate Hydrogels with Fast Degradability. Biomacromolecules.

[23] Exley S.E., Paslay L.C., Sahukhal G.S., Abel B.A., Brown T.D., McCormick C.L., Heinhorst S., Koul V., Choudhary V., Elasri M.O. (2015). Antimicrobial Peptide Mimicking Primary Amine and Guanidine Containing Methacrylamide Copolymers Prepared by Raft Polymerization. Biomacromolecules.

[24] Palermo E.F., Kuroda K. (2009). Chemical Structure of Cationic Groups in Amphiphilic Polymethacrylates Modulates the Antimicrobial and Hemolytic Activities. Biomacromolecules.

[25] Ergene C., Palermo E.F. (2017). Cationic Poly(benzyl ether)s as Self-Immolative Antimicrobial Polymers. Biomacromolecules.

[26] Paslay L.C., Abel B.A., Brown T.D., Koul V., Choudhary V., McCormick C.L., Morgan S.E. (2012). Antimicrobial Poly(methacrylamide) Derivatives Prepared via Aqueous RAFT Polymerization Exhibit Biocidal Efficiency Dependent upon Cation Structure. Biomacromolecules.

[27] Lee A.L.Z., Ng V.W.L., Gao S., Hedrick J.L., Yang Y.Y. (2014). Injectable hydrogels from triblock copolymers of vitamin E-functionalized polycarbonate and poly(ethylene glycol) for subcutaneous delivery of antibodies for cancer therapy. Adv. Funct. Mater..

[28] Chin W., Yang C., Wee V., Ng L., Huang Y., Cheng J., Tong Y.W., Coady D.J., Fan W., Hedrick J.L. (2013). Biodegradable Broad-Spectrum Antimicrobial Polycarbonates: Investigating the Role of Chemical Structure on Activity and Selectivity. Macromolecules.

[29] Tan J.P.K., Gao S., Hedrick J.L., Zhang Y., Guo X.D., Li L., Yang Y., Fukushima K., Wang H., Yang C. (2011). Biodegradable nanostructures with selective lysis of microbial membranes. Nat. Chem..

[30] Yang C., Krishnamurthy S., Liu J., Liu S., Lu X., Coady D.J., Cheng W., De Libero G., Singhal A., Hedrick J.L. (2016). Broad-Spectrum Antimicrobial Star Polycarbonates Functionalized with Mannose for Targeting Bacteria Residing inside Immune Cells. Adv. Healthc. Mater..

[31] Tan J.P.K., Coady D.J., Sardon H., Yuen A., Gao S., Lim S.W., Liang Z.C., Tan E.W., Venkataraman S., Engler A.C. (2017). Broad Spectrum Macromolecular Antimicrobials with Biofilm Disruption Capability and In Vivo Efficacy. Adv. Healthc. Mater..

[32] Qiang N., Yang W., Li L., Dong P., Zhu J., Wang T., Zeng C., Quan D. (2012). Synthesis of pendent carboxyl-containing poly(ε-caprolactone-co- β-malic acid)-block-poly(l-lactide) copolymers for fabrication of nano-fibrous scaffolds. Polymer.

[33] Alarçin E., Lee T.Y., Karuthedom S., Mohammadi M., Brennan M.A., Lee D.H., Marrella A., Zhang J., Syla D., Zhang Y.S. (2018). Injectable shear-thinning hydrogels for delivering osteogenic and angiogenic cells and growth factors. Biomater. Sci..

[34] Xu Y., Zhang K., Reghu S., Lin Y., Chan-Park M.B., Liu X.W. (2019). Synthesis of Antibacterial Glycosylated Polycaprolactones Bearing Imidazoliums with Reduced Hemolytic Activity. Biomacromolecules.

[35] Sano S., Tsumura T., Horibe M., Nakao M. (2013). Catalytic asymmetric ring-opening of σ-symmetric cyclic carbonates with chiral bronsted acid catalysts. Synlett.

[36] Bordes C., Fréville V., Ruffin E., Marote P., Gauvrit J.Y., Briançon S., Lantéri P. (2010). Determination of poly(ε-caprolactone) solubility parameters: Application to solvent substitution in a microencapsulation process. Int. J. Pharm..

[37] Woodruff M.A., Hutmacher D.W. (2010). The return of a forgotten polymer—Polycaprolactone in the 21st century. Prog. Polym. Sci..

[38] Li L., Wang Q., Lyu R., Yu L., Su S., Du F.S., Li Z.C. (2018). Synthesis of a ROS-responsive analogue of poly(ε-caprolactone) by the living ring-opening polymerization of 1,4-oxathiepan-7-one. Polym. Chem..

[39] Li L., Zhai H., Wang T., Qiu X., Qiang N., Dong P., Bai Y., Peng A.Y., Quan D. (2019). Bromine-functionalized poly(carbonate-co-lactide)s: Synthesis, characterization and post-polymerization functionalization. Polymer.

[40] Ong Z.Y., Coady D.J., Tan J.P.K., Li Y., Chan J.M.W., Yang Y.Y., Hedrick J.L. (2016). Design and synthesis of biodegradable grafted cationic polycarbonates as broad spectrum antimicrobial agents. J. Polym. Sci. Part A Polym. Chem..

[41] Ng V.W.L., Tan J.P.K., Leong J., Voo Z.X., Hedrick J.L., Yang Y.Y. (2014). Antimicrobial polycarbonates: Investigating the impact of nitrogen-containing heterocycles as quaternizing agents. Macromolecules.

