# Supplementary material for: Synthesis, Characterization, and Antimicrobial Evaluation of Random Poly(ester-Carbonate)s Bearing Pendant Primary Amine in the Main Chain
Source: Polymers (Basel). 2020 Nov 10;12(11):2640. doi: 10.3390/polym12112640 (PMC7696737; doi:10.3390/polym12112640)
Supplement: Supplementary file 1 [file polymers-12-02640-s001.pdf]

Synthesis, Characterization, and Antimicrobial Evaluation of Random Poly(ester-Carbonate)s Bearing Pendant Primary Amine in the Main Chain

Peng Dong, Jing Feng, Sujuan Li, Tingli Sun, Qingshan Shi * and Xiaobao Xie *

Guangdong Provincial Key Laboratory of Microbial Culture Collection and Application, State Key Laboratory of Applied Microbiology Southern China, Guangdong Institute of Microbiology, Guangdong Academy of Sciences

*Correspondence: jigan@gdim.cn (Q.S.); Tel.: +86-2087137652(Q.S.); xiexb@gdim.cn (X.X.); Tel.: +86-2037656986 (X.X.)

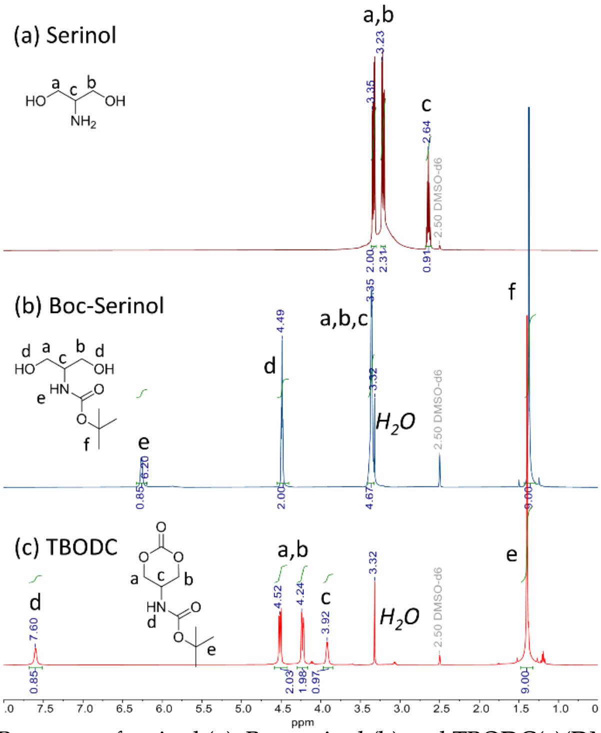

Figure S1. ^{1}H NMR spectra of serinol (a), Boc-serinol (b) and TBODC(c)(DMSO-d_6, 400 MHz).

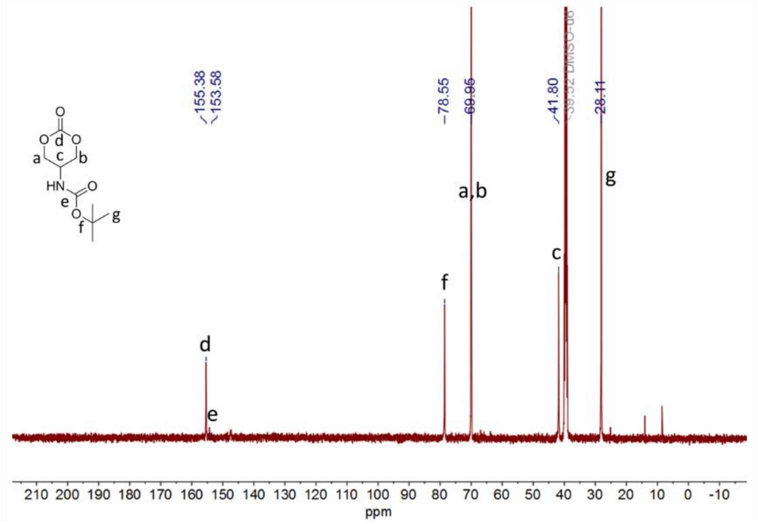

Figure S2. ^{13}C NMR spectrum of TBODC (DMSO-d_6, 100 MHz)

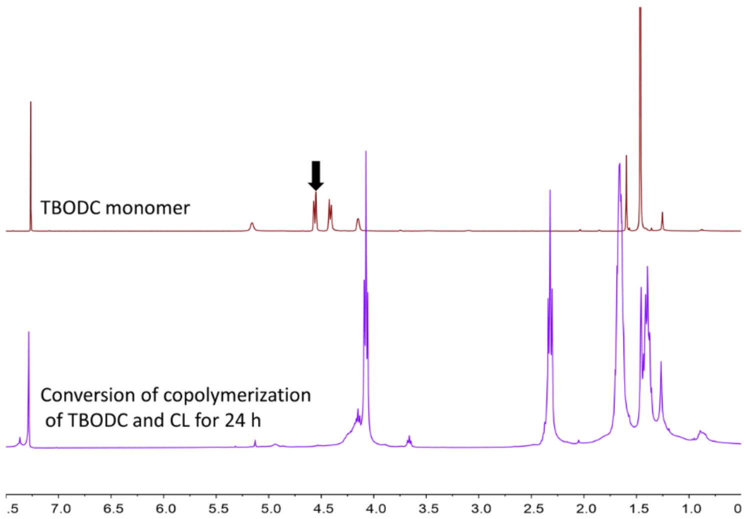

Figure S3. Investigation of copolymerization time of TBODC and CL.

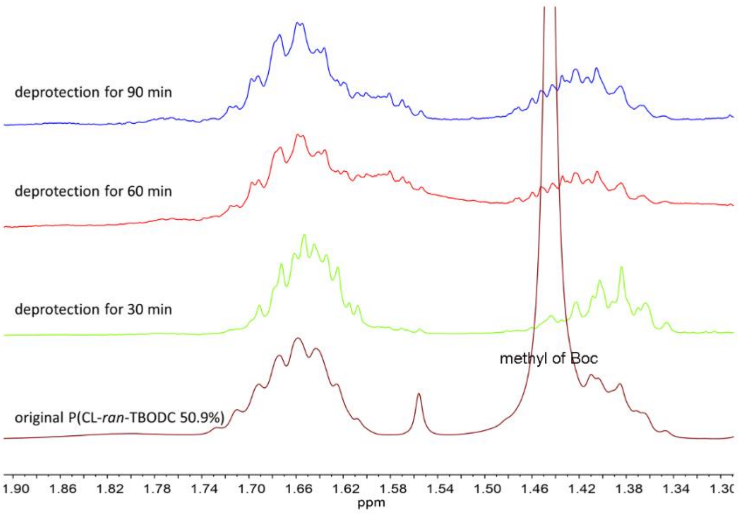

Figure S4. ^{1}H NMR spectra of P(CL-*ran*-TBODC 50.9%) after deprotection for certain time
